# Adaptation of High-Altitude Plants to Harsh Environments: Application of Phenotypic-Variation-Related Methods and Multi-Omics Techniques

**DOI:** 10.3390/ijms252312666

**Published:** 2024-11-26

**Authors:** Kai-Lu Zhang, Ya-Nan Leng, Rui-Rui Hao, Wen-Yao Zhang, Hong-Fei Li, Mo-Xian Chen, Fu-Yuan Zhu

**Affiliations:** 1 The Southern Modern Forestry Collaborative Innovation Center, State Key Laboratory of Tree Genetics and Breeding, Key Laboratory of State Forestry and Grassland Administration on Subtropical Forest Biodiversity Conservation, College of Life Sciences, Nanjing Forestry University, Nanjing 210037, China; 2 State Key Laboratory of Desert and Oasis Ecology, Xinjiang Institute of Ecology and Geography, Chinese Academy of Sciences, Urumqi 830011, China

**Keywords:** high-altitude plants, adaptation, transplant experiment, multi-omics techniques, environmental stress

## Abstract

High-altitude plants face extreme environments such as low temperature, low oxygen, low nutrient levels, and strong ultraviolet radiation, causing them to adopt complex adaptation mechanisms. Phenotypic variation is the core manifestation of ecological adaptation and evolution. Many plants have developed a series of adaptive strategies through long-term natural selection and evolution, enabling them to survive and reproduce under such harsh conditions. This article reviews the techniques and methods used in recent years to study the adaptive evolution of high-altitude plants, including transplantation techniques, genomics, transcriptomics, proteomics, and metabolomics techniques, and their applications in high-altitude plant adaptive evolution. Transplantation technology focuses on phenotypic variation, which refers to natural variations in morphological, physiological, and biochemical characteristics, exploring their key roles in nutrient utilization, photosynthesis optimization, and stress-resistance protection. Multiple omics technologies, including genomics, transcriptomics, proteomics, and metabolomics, have revealed genes, regulatory pathways, and metabolic networks associated with phenotypic variations at the genetic and molecular levels. At the same time, the limitations and deficiencies of current technologies used to study plant adaptation to high-altitude environments were discussed. In addition, we propose future improvements to existing technologies and advocate for the integration of different technologies at multiple levels to study the molecular mechanisms of plant adaptation to high-altitude environments, thus providing insights for future research in this field.

## 1. Introduction

With an average elevation of about 4000 m, the Qinghai–Tibet Plateau (QTP), commonly referred to as the Third Pole or the Roof of the World, is the biggest and highest plateau on earth [1]. Its geographical location is extremely special, and the environment is extremely diverse. It has typical environmental features such as low temperature, low oxygen levels, and intense ultraviolet radiation. Therefore, it is being used as a natural laboratory to study the adaptation and evolution of species in extreme environments [2].

To withstand the extreme natural environment of the plateau, QTP plants have evolved a number of special adaptive traits, such as distinct morphological traits of the root, stem, and leaf; plant interactions; pollinator interactions; petal color; reproduction; and resource allocation patterns [3]. Cushion plants, as a dominant plant in the QTP, have a compact growth pattern and a shorter plant height [4]. Perennial herbaceous plants found on the QTP have consistent leaf proportions, but their biomass allocation patterns are characterized by an increase in fine roots and a decrease in aboveground reproductive structures. Increasing the surface area of fine roots for absorption and maintaining biomass investment in photosynthetic structures helps high-altitude plants compensate for the carbon gain and reduced nutrient uptake at cold temperatures [5]. According to several studies [6,7,8,9], plants on the QTP have evolved specialized adaptive traits in their anti-stress physiological metabolism. For instance, enhancing the concentration of flavonoid compounds aids in resisting ultraviolet radiation, growing ephemeral shoots to completely avoid freezing, and increasing the leaf thickness and stomatal density to adapt to high light intensity and oxidative stress. High-altitude environments are mainly exposed to adversity stresses such as low oxygen, low temperature, and strong UV radiation. Therefore, compared with other extreme environments (e.g., arid or saline environments), the uniqueness of high-altitude adaptation is mainly reflected in low oxygen tolerance, UV resistance, and special metabolic pathways.

High-altitude plants usually have narrow distribution ranges and unique genetic resources, which are essential for maintaining ecosystem stability and function. Identifying their adaptive characteristics can provide a scientific basis for biodiversity conservation in these fragile ecosystems [10,11]. Many high-elevation plants play important roles in water regulation, soil stabilization, and carbon sequestration, services that are critical for downstream ecosystems [12,13]. With global climate change, crops are facing increasing stress from adversity. Plants at high altitude show unique advantages in adversity adaptation, such as resistance to cold, drought, and disease [14]. By studying the adaptive mechanisms of high-altitude plants, we can tap into and utilize these advantageous genetic resources to provide new ideas and approaches for breeding crops for stress tolerance, thus improving crop stress tolerance and yield stability [15,16,17,18]. In addition, by understanding how high-altitude plants survive in poor soils and extreme conditions, we can provide inspiration for sustainable agricultural practices in marginal lands [19,20]. Given the vulnerability of high-altitude ecosystems and the severe challenges of global climate change, in-depth research on the adaptive mechanisms of high-altitude plants is particularly important and urgent. This will not only help us to better understand and cope with these challenges but also provide a scientific basis and technical support for ecological conservation and agricultural development. However, the regulatory mechanisms of genes related to cold resistance, UV resistance, and low-oxygen adaptation in high-altitude environments are not yet clear, and the interactions between high-altitude plant community compositions, their distribution with the environment, and the potential of emerging technologies (such as remote sensing and artificial intelligence) in monitoring high-altitude plant growth and health status have not been fully explored, revealing significant gaps in applying genomics and bioinformatics to analyze the adaptation and evolution of high-altitude plants.

Emerging technologies are being used in high-altitude ecosystem studies in addition to conventional techniques such as ecological surveys, physiological experiments (e.g., greenhouse experiments, garden experiments), geochemical analysis, and genetic investigations. This paper reviews the progress made in conventional garden experiments and reciprocal transplant trials while also outlining the application of cutting-edge technologies to comprehensively understand vegetation ecology, species distribution, ecological functions, and adaptation mechanisms in high-altitude regions. The multi-omics approach has played a key role in revealing the mechanisms of energy consumption in organisms and their responses to environmental stress. This paper reviews relevant studies on high-altitude adaptation using omics techniques (Table 1). For high-altitude non-model plants, the de novo assembly technique not only lays the foundation for decoding the genome, but it also provides key data for analyzing the mechanisms of adaptation to extreme environments. By integrating transcriptomics and proteomics, these methods can identify genes that are subject to positive selection pressure under specific environmental conditions, deepening our understanding of how organisms adapt and respond to environmental changes through gene regulatory mechanisms. We discuss the limitations of current techniques for studying plant adaptation to high-altitude environments and make suggestions for improving existing methods.

## 2. Transplant Experiment

To adapt to environmental changes, populations of plants engage in plastic response, migration, and genetic adaptation through selection [21,22]. The most significant research areas in evolutionary genetics and adaptive evolution are phenotypic plasticity and genetic variability, which play a significant role in defining a species’ capacity for adaptation [23]. In short-term climate change, phenotypic plasticity can provide rapid adaptive responses, such as plants responding to temperature changes and water stress by adjusting growth patterns, flowering times, or physiological characteristics. As environmental changes continue, genetic adaptability will gradually accumulate and be fixed in the population, ensuring that plants can cope with the constantly changing climate over multiple generations [24]. Therefore, characterizing the adaptive potential of alpine plant species first involves studying a series of traits associated with plant adaptation and altitude adaptation.

**Figure 1:**
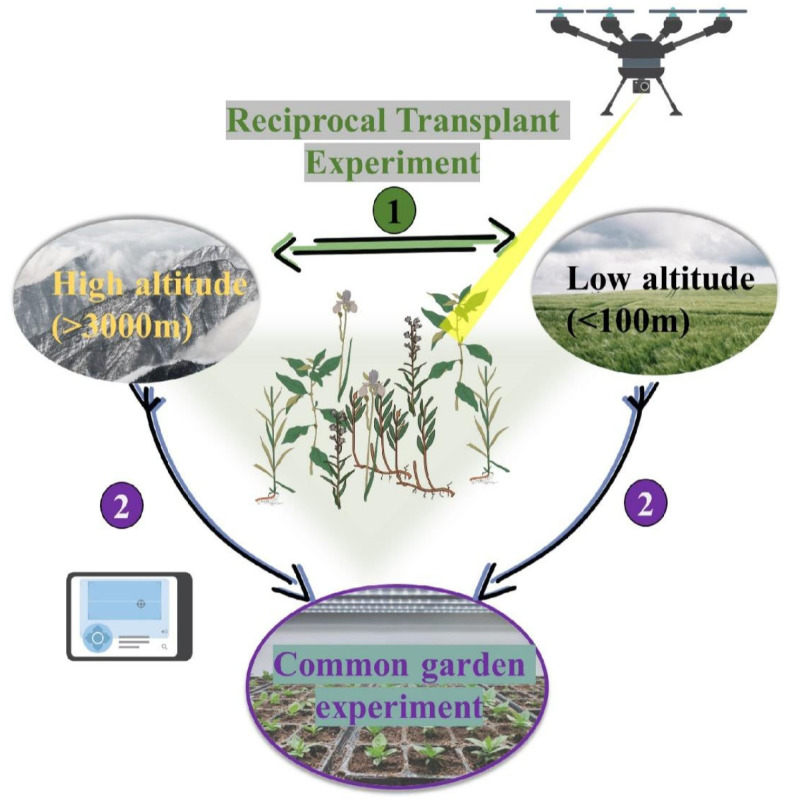
A diagram of the common garden experiment and the reciprocal transplant experiment. The common garden experiment (represented in purple) and the reciprocal transplant experiment (represented in green) are two common methods for studying plant adaptation. In the common garden experiment, seeds or plants from different altitudes are planted under the same standard environmental conditions, such as the same soil type, light exposure, temperature, and humidity. By comparing the growth and performance of plant species from different altitudes under the same environmental conditions, researchers can evaluate the differences in adaptability between different populations. In the reciprocal transplant experiment, seeds or plants from different altitudes are transplanted to altitudes outside of their original source. By comparing the growth and performance at their original source and at the transplanted locations, researchers can assess the adaptability and survival ability of different populations in different environments. Furthermore, precision agricultural technologies such as drones and sensor networks can be used to monitor the growth environment and physiological characteristics of medicinal plants, assisting growers in managing plant cultivation and growth processes more scientifically. The direction of the arrow indicates where the plant was transplanted.

The study of plant phenotypes in the process of adaptive evolution is mainly carried out in transplant experiments (also called field experiments) (Figure 1), which include common garden experiments and reciprocal transplant experiments [25,26]. These transplant experiments are classic tools for studying adaptive differentiation, which aims to describe the differentiation of phenotypes between populations derived from different sizes and geographic scales in order to better understand the phenotypes associated with adaptation and the selection pressures that influence them. The difference is that common garden experiments can compare the growth and other phenotypic traits of plant populations from different environments [26,27] to assess the genetic basis of population adaptation to different environments without considering environmental changes [28,29,30]. Therefore, common garden experiments have been widely used in forest trees to detect the adaptive signals of traits, including life history, phenology and allometric relationships [31,32,33,34]. When there is evidence that environmental differences between populations may drive adaptive genetic differentiation, the genetic mechanisms of genetic differentiation and adaptation can be further investigated by controlling for environmental variability in a common garden [35]. Through a common garden experiment, the study examined the differences in protein accumulation and physiological responses among four populations of *Myrsine coriacea* from different altitudes and assessed whether these differences exhibit genetic fixation [36]. The common garden experiment highlighted that the proteomic and physiological changes in the high-altitude population were due to local adaptation and abiotic selection pressures, even under common garden conditions. These studies showed that common garden experiments play a crucial role in understanding local adaptations to abiotic stress.

The reciprocal transplant experiment can directly detect local adaptation and the factors affecting adaptive differentiation of populations [31]. Through reciprocal transplants, local adaptation of a plant population can be demonstrated if it survives and grows best in its native environment and outperforms other populations of the species that have evolved in different environments [26]. Therefore, the quantification of local adaptation by reciprocal transplant experiments is still a common method to detect local adaptation in natural populations [27,31,37]. For example, in slope vegetation restoration experiments conducted in Jiuzhaigou County, Sichuan Province, to address the harsh environmental conditions in high-altitude areas, the research team employed film mulching technology. This method holds immense potential as it facilitates plant growth and maintains soil fertility. Experimental results revealed that under different coverage rates, film mulching treatment significantly enhanced plant growth and the soil nutrient content while reducing the plant density per unit area. In particular, at a coverage rate of 90%, both plant growth and the soil nutrient content were at their maximum. Although the rapidly changing environmental conditions in high-altitude areas may affect the effectiveness of membrane covering, such experiments offer reliable methods for addressing these challenges [38].

At present, many studies have applied common garden experiments and reciprocal transplant experiments to study the adaptive mechanism of plants in high-altitude areas such as the QTP [39,40,41,42,43,44,45]. These studies suggest that inheritance-based trait differentiation and altitude adaptation are common in plants. Many of these traits have moderate to high heritability [30]. Transplant experiments have the advantage of being simple and not requiring a lot of technology, but they may require a lot of time and labor, and the number of tested plants is usually relatively limited. Hyperspectral imaging technology effectively captures subtle changes in plant phenotypes. By using hyperspectral cameras mounted on an unmanned aerial vehicle (UAV) [46], a non-destructive method can be employed to assess the severity of stem rust in different wheat genotypes at very low infection levels, serving as a substitute for trained personnel in large-scale field disease surveillance. Furthermore, since plant phenotypes are dynamic and spectral similarities may exist, most phenotype data across different studies are difficult to compare using standardized methods. Remote sensing technology, based on deep learning models, can distinguish four different sugarcane varieties [47]. These technologies provide new opportunities for research on plant adaptation at high altitudes.

For example, satellite remote sensing can provide wide-area coverage, while UAV remote sensing can provide complementary observations under unfavorable conditions such as cloud cover [48], and ground-based phenotyping detects dynamic plant growth through various sensor technologies [49]. By integrating multiple data sources such as satellite remote sensing, UAV remote sensing, and ground-based observations, the spatial and temporal resolution and accuracy of the data can be improved. In addition, current advanced artificial intelligence techniques can improve data precision and accuracy [50]. For example, deep learning and machine learning algorithms can be used to automate the processing and analysis of large-scale remote sensing data [48,51]. These algorithms can identify plant species-specific features, track changes in plant communities, and even predict future trends in ecological functions.

On the other hand, Artificial Intelligence (AI) is used to analyze plant health in real time (e.g., photosynthesis efficiency, vegetation index) and to predict future trends through modeling, such as the long-term adaptability of plants to environmental changes. However, the application and popularity of these technologies are still far from adequate at high altitudes, and there are still challenges such as data transmission and equipment maintenance. The efficiency of data collection and transmission can be optimized through the development of low-power remote sensing devices and by reliance on satellites or other long-term observatories. The interpretability of the AI algorithms used should be ensured so that ecologists can understand the output of the models and thus make sound ecological judgments. Collaborating with relevant institutions and universities can establish a dedicated monitoring and evaluation platform to provide functions such as data sharing, algorithm optimization, and results presentation. The platform can be open to researchers, administrators, and the public to promote the in-depth development of high-altitude plant adaptation research.

## 3. Genomics Facilitates the Molecular Basis of Adaptive Evolution

The plant genome governs the intricate responses and adaptive behaviors of plants across diverse environments. Species evolve based on genomic changes. The rapid advancement of high-throughput sequencing technologies has markedly enhanced sequencing throughput and quality while concurrently reducing costs. This progress has greatly accelerated genomic research and functional analysis [50]. Understanding genomic alterations is crucial for elucidating the molecular basis of morphological and physiological enhancements during plant evolution. In other words, plant genomes form the foundation for comprehending the adaptive evolution of plants in the plateau. However, research on plant molecular biology and genomics in the plateau lags behind that of vertebrates and other terrestrial plants. As a result, genome sequencing technology plays a vital role in studying plants in high-altitude regions, and it complements common garden experiments, jointly providing strong support for revealing the molecular mechanisms of plant adaptation to extreme environments.

Advances in genome assembly techniques have led to an increasing number of high-quality reference genomes being published. Since the genome construction of the model plant *Arabidopsis thaliana* [52], with the continuous innovation of high-throughput sequencing technology, more and more high-altitude plant genomes have been reported [51]. In recent years, the extensive collection of plant species and the assembly and resequencing of high-quality whole genomes in the plateau have greatly promoted research on the molecular genetics, phenotypic variation, and adaptive evolution of high-altitude plants [53]. For instance, analysis based on the genome sequence of *Crucihimalaya himalaica* revealed that dramatic changes in gene families in the signs and size of the positive selection of genes that could be candidates for *C. himalaica*’s adaptation to the harsh environments in the QTP [54]. High-altitude plants may have enhanced adaptive capacity through genome duplication, chromosome rearrangements, or segment deletions. Genomic studies can reveal how these structural variations affect a plant’s ability to adapt to its environment. For example, the assembly of the genome and the deciphering of whole-genome duplication (WGD) events of the tetraploid genome of *Rhododendron nivale* subsp. *boreale* have laid the foundation for the evolution and adaptation of *Rhododendrons* and provided valuable resources [55]. In addition, two chromosome-scale genomes of two *Eutrema* species have been assembled, which are from the high-altitude *E. heterophyllum* from the eastern QTP and *E. yunnanense* from lowland [56]. According to the comparative analysis of the two species, the results reveal the expansion of disease-resistance R genes (NBS-LRRs or NLRs) and genes related to DNA damage repair as well as cold-tolerant alpine plants, which show that the genomes of the two *Eutrema* reported are important genetic resources for the adaptative evolution of alpine plants across the world [56]. Moreover, a comparative study of *Prunus* genomes revealed that a striking expansion of the SINE retrotransposons had occurred in the genomes of Tibetan species, which promoted the accumulation of beneficial metabolites of the Tibetan *Prunus* species for adapting to the harsh environment [57]. A comparison of the genomes of several alpine plants as well as their lowland relatives revealed that some genes involved in reproduction and respiration (e.g., *MMD1*, *NBS1*, and *HPR*) may underlie the convergent adaptation of alpine plants to extreme environments [58]. In summary, based on comparative genomics, we can localize regions of selective signals associated with high-altitude acclimatization using methods such as the selective scanning of genomic data from plant populations at different altitudes. For example, certain gene families may exhibit strong positive selection signals, indicating their important roles in high-altitude environments.

The goal of population genetics is to identify evolutionary forces, such as natural selection, gene flow, and demographic fluctuations, that drive plant adaptation to local environments [59]. The increased accessibility of genome-wide data is transitioning population genetics into population genomics, revolutionizing our understanding of local adaptation [60,61]. Population and landscape genomic studies of adaptive evolution typically use sequence data generated by the whole genome, candidate genes, transcriptome, etc. to detect genetic variations in natural populations within an area or an entire species. Genomic signals selected in natural populations can be revealed by various genomic analysis methods, such as FST methods and environmental association analysis, which detect inter-population differentiation or associations between genetic variations and environmental gradients [23,28,62]. Recently, a chromosome-scale genome of *Ilex polyneura* in southwest China was assembled, and by conducting a population genomics study, 34 candidate genes were found to have functions connected to responses to biotic and abiotic stresses under selection at various elevations [63].

However, several unavoidable disadvantages exist when employing population genetics or genomics approaches. For instance, identifying rare alleles crucial for local adaptation can be challenging, especially in complex demographic contexts with strong gene-flow episodes [26]. Recently, landscape genomics has been a useful complementary method for determining the adaptive loci responsible for local adaptation [28,64,65,66]. By combining approaches from landscape genomics (Bayenv2) and population genomics (BayeScan), two hotspot regions that had strong natural selection signals linked to sun radiation and altitude were identified. These regions contained 14 genes primarily involved in abiotic stress resistance and effective reproduction [59]. High-altitude and alpine plants are ideal materials for studying adaptive evolution. Increasing evidence indicates that population genomics and landscape genomics significantly contribute to the study of plant adaptive evolution [59,67]. Additionally, applying a multiscale landscape genomic approach can detect selection signatures along environmental gradients such as solar radiation, wind exposure, and altitude, indicating that adaptive pressures may be driven by fine-scale topography [67]. Population and landscape genomic approaches study adaptive evolution by detecting genetic variations, which are crucial for understanding how to manage and protect species populations under changing climate conditions.

## 4. The Role of Multi-Omics in the Adaptive Evolution of Alpine Plants

**Figure 2:**
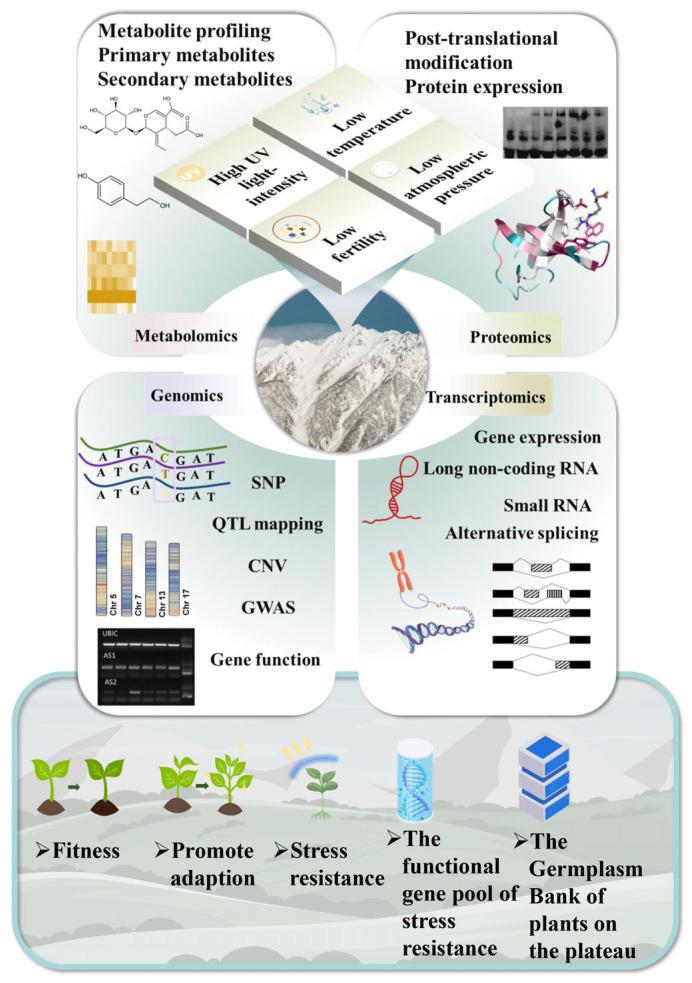
Omics technology research into plant adaptation to high altitude. In high-altitude areas, the environmental conditions, including high UV intensity, low fertility, low atmospheric pressure, and low temperatures, pose significant challenges. To address these challenges, current omics technologies offer multiple research perspectives. Metabolomics, through the analysis of metabolite spectra, primary metabolites, and secondary metabolites, reveals the metabolic adaptation mechanisms of organisms in high-altitude environments. Proteomics focuses on post-translational modifications and changes in protein expression levels, providing insights into the functional adaptations of proteins to high-altitude environments. Genomics investigates single-nucleotide polymorphisms, quantitative trait loci, copy-number variations, and other genetic aspects, revealing genetic adaptation strategies of organisms to high-altitude environments. Transcriptomics explores gene expression regulation, long non-coding RNAs, small RNAs, and other aspects, elucidating the transcriptional regulatory mechanisms of organisms in high-altitude environments. Future research will focus on enhancing biological adaptability, promoting adaptation processes, expanding the gene pool for stress resistance and stress resistance, and exploring medicinal plant resources. Additionally, efforts will be made to establish a high-altitude plant germplasm repository to support the conservation and utilization of biological resources in high-altitude areas. The experimental figures are quoted from previously published articles [68,69,70,71].

At present, research on alpine plant adaptation primarily focuses on molecular aspects, revealing some genes, proteins, and metabolites associated with cold resistance, ultraviolet resistance, and hypoxia adaptation. Different omics technologies have their own characteristics in terms of breadth and depth. Based on the four levels of “What possibly happens”, “What probably happens”, “What happened”, and “What actually happens”, genomics, transcriptomics, proteomics, and metabolomics can each be used to validate gene expression, protein transcription and modifications, and changes in various metabolic levels. Therefore, establishing an integrated adaptive model can validate and help to understand the adaptation of alpine plants in a multi-level, systematic, and comprehensive manner. By integrating data, it becomes possible to more effectively identify key gene–protein–metabolite interaction pathways and to construct core models of adaptation mechanisms under environmental stress. Moreover, multi-omics integration can mitigate the limitations of single-omics analyses, providing a more comprehensive and accurate perspective. Despite the increasing number of high-altitude plant genomes being reported, a substantial number of plants still lack reference genomes. Consequently, investigating gene expression evolution, gene regulation, and species adaptation through other omics approaches (Figure 2), such as transcriptomics, becomes particularly important.

Transcriptomics provides valuable insights into the adaptive evolution of high-altitude plants by studying changes in gene expression in plants under different environmental conditions [72]. Differences in adaptation mechanisms can be revealed by comparative transcriptome analysis. It was found that hundreds of positively selected genes (PSGs) were identified in high-elevation *Saussurea* species through comparative transcriptome analysis of five *Saussurea* species from different elevations; among these, *RAD54*, *RUP2*, and *TT5* are involved in DNA repair, the response to UV-B, flavonoid biosynthesis, and the cell response to hypoxia. Some important alpine-species-specific gene families were detected by phylogenetic cluster analysis, which may contribute to the defense against hypothermia and hypoxia in the Tibetan Plateau [73]. The same method was applied to a comparative analysis of two endangered high-alpine herbal plants endemic to China, *Notopterygium incisum* and *Notopterygium franchetii* (Apiaceae), and 18 candidate PSGs, corresponding to the functional groups of RNA splicing, were found that were involved in high-altitude adaptation and binding to RNA-binding protein (RBP) [74,75]. In the application of this technique, a number of precedents were carried out to study non-model plants. For example, high-quality de novo assembly of *Potentilla bifurca* from two altitudes (1725 masl and 3215 masl) was reported for the first time, and pathway analysis revealed a large number of DEGs encoding key enzymes involved in secondary metabolites, including phenylpropane and flavonoids [76].

Transcriptomics can also identify many key genes related to adaptation by analyzing the gene expression profiles of high-altitude plants under specific environmental conditions. For example, for the first time, according to the altitudinal adaptation mechanism of different-color flowers of alpine *Rhododendron* L. along the altitudinal gradient, specific PSGs that may be closely related to their evolution were identified, such as *MEKK1*, *YODA*, *PP2C*, and *SnRK2* in *R. fastigiatum*. These four PSGs are significantly enriched in the plant *MAPK* signaling pathway, while *MEKK1* and *YODA* are closely related to plant stress signal transduction [77]. In addition, based on transcriptome data of the alpine plant *Saussurea obvallata*, genes related to cuticlar waxes (*CER1*, *CER3*, *CER4*, and *MAH1*) and flavonoid biosynthesis pathways (*4CL*, *CHS*, *CH1*, *F3-H*, *TT7*, and *OMT*) were more highly expressed in leafy bracts than in other leaf tissues, and changes of these pathway genes may be important strategies for alpine plants to adapt to the high-altitude environment [58]. These transcriptome data all reveal, at the molecular level, how plants on the Tibetan Plateau survive in extreme environments [73,78,79,80]. The abundance of reference transcriptome data enables the analysis of specific gene families, candidate genes, and regulatory pathways in the Tibetan Plateau environment, with or without reference genomes. This provides valuable genomic resources and a basis for further studies on the molecular adaptation mechanism of plateau plants. However, transcriptomics is often based on mixed samples (such as entire tissues or organs) and lack details about the different cell types and spatial distribution within cells. Therefore, we propose the use of spatial transcriptome technology to help determine the expression of specific adaptive genes in the leaves, roots, and stems of plants at high altitudes so as to reveal the cooperative adaptation mechanism between different organs of plants [81,82].

Proteomic analysis is a powerful tool for studying the dynamic protein profiles of plants in response to high-altitude stress conditions [83]. Extreme environments alter cellular homeostasis, leading to changes in the final function of plant proteins. Compared with the genome and transcriptome, the proteome is more phenotypic and more directly responsive to environmental stress, which can provide a comprehensive and in-depth perspective on the mechanisms of plant adaptation to alpine environments [84,85]. Proteomic analysis of the accumulation of and changes in various stress-responsive proteins (18.1 class I HSP, HSP 70 family, heat shock factor binding protein, ATP synthase delta chain, and so on), enzymatic antioxidants, and the primary metabolism of *Picrorhiza kurroa* in the Himalayan region revealed their ability to cope with environmental changes along with the altitudinal gradient [86]. But the unique environment at high altitudes provides difficulty for the protein extraction and analysis process of alpine plants, as well as increasing the diversity of protein post-translational modifications, making experiments and data analysis more difficult. In addition, proteomics faces the challenge of detecting low-abundance proteins. In order to overcome these shortcomings, the method of multi-omics integration analysis has been used to make the protein data more reliable. Using comparative proteomics, we studied the dynamic patterns of protein expression in the alpine plant *Lamiophlomis rotata* located at three different altitudes (4350, 4800, and 5200 m) and identified 84 different protein spots. They demonstrated that Hydrogen sulfide (H_2_S) can activate antioxidative enzymes and GSNOR (S-nitrosoglutathione reductase), thereby reducing ROS (reactive oxygen species) and RNS (reactive nitrogen species) levels and triggering a range of downstream defense responses, including protein degradation, proline and soluble sugar biosynthesis, and HSP accumulation [87]. Similarly, using comparative proteomics combined with physiological data, it was demonstrated that *Potentilla saundersiana* could enhance tolerance to high-altitude environments through a variety of pathways. For example, it regulates the root structure, leaf phenotype, photosynthetic capacity, and cell-wall structure; increases the contents of proline, soluble sugar, and flavonoids; and enhances the abundance of Autophagy protein 5a and SWI/SNF complex subunit SWI3A in response to environmental stress [88]. Using proteomics, functional proteins can be identified, post-translational modifications can be analyzed, and protein interaction networks can be constructed, providing key clues for the study of plant adaptation at high altitude. These studies not only deepen our understanding of the molecular mechanisms of plant adaptation to extreme environments but also provide a scientific basis for the development of stress-tolerant plants and the response to climate change.

Non-targeted metabolomics makes up for the shortcomings of proteomics in capturing immediate plant responses to environmental changes and comprehensively detecting metabolites. It can quickly reflect the physiological state of plants and provide extensive metabolic information [89,90]. For example, in order to reveal the adaptation strategy of *Picrorhiza kurroa* along the altitude environment, LC-MS-based non-targeted metabolite analysis and targeted analysis of sugars, amino acids, bitter glycosides and their corresponding phenolic acids were performed using leaves, roots and rhizomes. Sugar accumulation is believed to contribute to plant tolerance by acting as osmolytes and stabilizers of proteins and membranes. The accumulation of amino acids under stress may be related to nitrogen storage and serve as precursors for the synthesis of secondary metabolites (SMs). The role of bitter glycosides remains largely unclear, but their differential accumulation could be hypothesized to play a role in plant adaptation, and the results show that the content of picroside (I–IV) vary significantly in an organ-specific manner. The regulation of sugar and amino acid metabolism, remodeling of the root cell wall, ascorbate biosynthesis and cleavage, occurrence of a partial TCA (tricarboxylic acid) cycle, purine catabolism and the salvage route, pyrimidine biosynthesis, alteration of the lipid composition, and inhibition of gibberellins and cytokinin can effectively promote the adaptation of high-altitude *Picrorhiza kurro* [91]. Metabolomics studies revealed that high-altitude environmental conditions were important factors in inducing changes in secondary metabolites in Hemp (*Cannabis sativa* L.) inflorescence. Plants growing at high altitudes showed a higher total amount of terpenes, of which β-myrcene, *trans*-caryophyllene and α-humulene were the main contributors. In addition, the study also showed that the UV-length-exposure environment is conducive to the production of CBDA (cannabidiolic acid) and cannaflavins. Different types of flavonoids are involved in plant protection mechanisms, particularly through their free-radical scavenging activity and their ability to filter short-wavelength UV-B radiation [92]. Using pseudotargeted metabolomics analysis, it was found that in order to adapt to the strong ultraviolet radiation and low-temperature stress conditions in the high-altitude environment, phenylpropanoid biosynthesis, phenylalanine, tyrosine and tryptophan biosynthesis and phenylalanine metabolism related to flavonoid biosynthesis in *Draba oreades* Schrenk plants showed an up-regulated trend [93]. The advantage of metabolomics over other omics is that genomic information is not required. It has been shown that changes in primary metabolites such as sugars and amino acids are adaptive strategies for high-altitude plants, and that reprogramming of these metabolites is responsible for the accumulation of secondary metabolites such as phenolics and flavonoids, which are responsible for the adaptation of plants to high-altitude conditions [14].

The multi-omics analysis more systematically reveals the adaptation mechanism of alpine plants in extreme environments. In studying the adaptation of *Sinopodophyllum hexandrum* to a high-altitude environment, proteomic, transcriptomic, and metabolomic analyses revealed significant increases in the flavonoid, flavonol, and anthocyanin content in plants under conditions of 3300 m altitude and high light intensity. Several differentially expressed proteins (DEPs) and differentially expressed genes (DEGs) involved in the light response and flavonoid biosynthesis were identified at high altitudes, including up-regulation of *PAL*, *CHS1*, *IFRL*, *ANS*, *MYB4*, *BHLH137*, *CYP6*, *PPO1ERF5*, *HSP18.1*, *HSP70*, *UBC4*, *ERF5*, *ERF9*, *APX3*, and *EX2*. This suggests that high light intensity may enhance the accumulation of flavonoids, thereby improving podophylla’s adaptability to high altitude and high light intensity [94].

In summary, multi-omics analysis provides important molecular evidence for studying the adaptability of alpine plants under different altitudinal gradients. These results have laid a solid foundation for revealing the molecular and physiological mechanisms of alpine plant adaptation to stress conditions in the future. In theory, it is entirely possible to transfer the adaptive characteristics unique to high-altitude plants, such as cold resistance, drought resistance, and high UV resistance, to crops using genetic engineering and traditional breeding techniques [95]. These technologies provide scientists with a means of directly manipulating biological genetic information, making it possible to transfer genetic material across species or varieties. However, achieving this goal requires interdisciplinary collaboration, in-depth fundamental research, and strict regulatory measures to ensure safe application of the technology.

## 5. Conclusions and Perspectives

Overall, we have gained some insights into the molecular and physiological mechanisms of acclimatization in high-altitude plants through the research methods and techniques described above. For example, high-altitude plants enhance low-temperature tolerance by synthesizing antifreeze proteins and by changing the fatty acid composition of cell membranes. These changes help plants maintain cell membrane integrity and stability under low-temperature conditions. In terms of their physiological response, high-elevation plants cope with low-temperature stress by adjusting their growth cycle and increasing biomass accumulation, thereby improving survival and reproduction. In addition, high-altitude plants can protect themselves against intense UV radiation by synthesizing UV-absorbing substances such as flavonoids and phenylpropanoid compounds. These substances absorb and scatter UV rays, thereby protecting plant cells from damage. Physiologically, high-elevation plants reduce UV exposure by increasing the leaf thickness and decreasing the leaf area, thereby increasing their resilience to intense UV radiation. However, high-elevation plants are usually exposed to the superimposed effects of multiple environmental stresses (e.g., low oxygen, low temperature, and intense UV radiation). However, our understanding of how these stresses interact and how they work together to affect plant acclimatization remains limited. Future studies need to explore in greater depth the interactions among these stresses and their combined effects on plant adaptive networks.

At present, the establishment of genetic transformation systems and plant tissue culture technologies suitable for high-altitude plants is a major technical challenge. At high altitudes, many rare medicinal plants face reproductive problems, such as non-mode propagation, long cycles, conversion technology bottlenecks, and genetic hybridization difficulties [96]. Establishing suitable genetic transformation systems and plant tissue culture techniques is a major technical challenge for cultivating high-altitude plants. Applying the adaptation mechanisms of alpine plants to agricultural breeding, particularly for traits such as cold resistance and UV tolerance, holds great promise. Wild plants, shaped by natural selection, have more diverse and complex adaptation mechanisms. In contrast, crops, which are typically subject to artificial selection and improvement, exhibit strong adaptability and high productivity. Through genetic improvement, combined with multi-omics technologies (such as genomics, transcriptomics, etc.), key genes related to traits like cold resistance and UV tolerance can be identified, providing innovative insights for crop breeding. Today, modern breeding methods, such as gene transformation and gene editing, are evolving rapidly, offering strong support for the development of this field. The most common methods of plant genetic transformation include *Agrobacterium*-mediated transformation and gene gun [97,98], as well as the pollen tube pathway and ultrasonic methods. Recently, the application of nanomaterials in genetic transformation has shown great potential. Common nanomaterials include carbon nanotubes, LDH, gold nanoparticles, carbon dots, and peptide carriers [70,71,99,100,101]. Nanomaterials can penetrate cell walls without external force and are not restricted by plant species. They can also carry large amounts of nucleic acids and protect them. Although this technology is still in its early stages, with unclear mechanisms and potential bioaccumulation concerns in medicinal plants, its unique advantages in design and modification make it an efficient method for developing stable genetically transformed regenerating plants, especially for high-altitude species. To address the cumbersome tissue culture process in genetic transformation, Dr. Zhu’s team developed a cut–dip–budding (CDB) delivery system. The system takes advantage of the plant’s “budding” ability, eliminating the need for tissue culture [102]. The system has been tested on *Taraxacum kok-saghyz*, *Coronilla varia*, *Ailanthus altissima*, *Aralia elata*, and *Clerodendrum chinense*, which were previously difficult or impossible to transform. It now enables the successful transformation or gene editing of these species. Although the validation is limited to plants with tillering ability, its application in high-altitude plants is promising [103].

**Table 1:** Case studies of omics approach for high-altitude plant adaptation.

Plant Species	Altitude	Omics Approach	Effect	Reference
*Crucihimalaya himalaica*	Seedlings of *C. himalaica* were sampled from an altitude of 4010 m.	Genomic	Gene families showing dramatic changes in size and genes showing signs of positive selection are likely candidates for *C. himalaica*’s adaptation to intense radiation, low temperature, and pathogen–depauperate environments in the QTP. Loss of function at the S-locus, the reason for the transition to self-fertilization of *C. himalaica*, might have enabled its QTP occupation.	[54]
*Prunus* sp.	A total of 377 accessions of *Prunus* germplasm along altitude gradients ranging from 2067 to 4492 m in the Himalayas were collected and sequenced.	Genomic and metabolomic	A total of 379 metabolites had significant genetic correlations with altitudes; in particular, phenylpropanoids were positively correlated with altitudes. Specific SINE insertions change the expression of altitude-related genes.	[57]
*Saussurea* sp.	Three (*S. pachyneura*, *S. salwinensis*, and *S. velutina*) were collected between 4550 and 4620 m, and the other two (*S. amurensis* and *S. amara*) were collected between 150 and 350 m	Transcriptomic	Gene families specific to alpine species were identified, which involve oxidoreductase activity, pectin metabolism, lipid transport, and polysaccharide metabolism, potentially aiding in the defense against hypoxia and the freezing temperatures of the QTP. Also, hundreds of genes under positive selection were discovered, related to DNA repair, membrane transport, UV-B and hypoxia responses, reproduction, and nutrient metabolism, likely contributing to *Saussurea*’s adaptation to high-altitude environments.	[73]
*Potentilla bifurca*	Sample selected from two altitude ranges −3215 and 1725 masl	Transcriptomic	Fifty differentially expressed genes (DEG), including peroxidase, superoxide dismutase protein, and the ubiquitin-conjugating enzyme responded to abiotic stresses; a large number of DEGs encode key enzymes involved in secondary metabolites, including phenylpropane and flavonoids; 298 potential genomic SSRs were identified for genetic diversity assessment.	[76]
*Rhododendron* sp.	Four colored species, *Rhododendron fastigiatum* Franch (4194 m), *Rhododendron lacteum* Franch (3927 m), *Rhododendron facetum* I. B. Balfour & Kingdon Ward (2817 m) and *Rhododendron pachypodum* Balf. f. et W. W. Smith (2413 m), were collected.	Transcriptomic and metabolomic	Genes related to carbohydrates, fatty acids, amino acids and flavonoids biosynthesis play important roles in the altitude adaptability.	[77]
*Potentilla saundersiana*	Samples from 4350 to 5200 m in altitude.	Proteomic	Proteins involved in antioxidative activity, primary metabolites, epigenetic regulation, and protein post-translational modification play important roles in conferring tolerance to alpine environments.	[88]
*Sinopodophyllum hexandrum*	Leaves from 3-year-old plants were collected from 3300 and 2300 m.	Proteomic and transcriptomic	Nine DEPs and 41 DEGs were identified as being involved in flavonoid biosynthesis and the light response at 3300 m.	[94]
*Herpetospermum pedunculosum*	Samples from 2800 m, 3000 m, 3100 m and 3300 m.	Proteomic	High level of expression of some proteins, such as oxygen-evolving enhancer proteins, calreticulins, and S-adenosyl-l-homocysteine hydrolase, might confer greater tolerance in *H. pedunculosum* to the complex environment associated with high altitudes.	[104]
*Cannabis sativa*	Raw inflorescences material obtained from plants cultivated in 1200 m and 130 m altitude.	Metabolomic	All plants grown at altitude exhibited a higher total amount of terpenes when compared with plains counterparts, with β-Myrcene, trans-Caryophyllene and α-Humulene as the main contributors.	[92]
*Draba oreades*	Samples were collected at altitudes of 3800 m, 4000 m and 4200 m.	Metabolomic	Phenylalanine, tyrosine, and tryptophan biosynthesis and phenylalanine metabolism related to the biosynthesis of flavonoids were up-regulated in the high-altitude group, and 10 important metabolites were identified as potential biomarkers.	[93]
*Cyclocarya paliurus*	Mature leaves with the largest leaf area at F4 stage from 280 m and 920 m.	Metabolomic and transcriptomic	High altitude induces more flavonoid accumulation than low altitude, which may be contributed by the up-regulation of genes involved in energy and protein synthesis.	[105]
